# From Hollow to Solid Carbon Spheres: Time-Dependent Facile Synthesis

**DOI:** 10.3390/nano8100861

**Published:** 2018-10-20

**Authors:** Wojciech Kukułka, Karolina Wenelska, Martyna Baca, Xuecheng Chen, Ewa Mijowska

**Affiliations:** Nanomaterials Physicochemistry Department, Faculty of Chemical Technology and Engineering, West Pomeranian University of Technology, Szczecin, Piastow Av. 45, 70311 Szczecin, Poland; Karolina.Wenelska@zut.edu.pl (K.W.); martyna.baca@zut.edu.pl (M.B.); Xuecheng.Chen@zut.edu.pl (X.C.)

**Keywords:** mesoporous materials, carbon spheres, hollow carbon spheres, solid carbon spheres, CVD process, time dependence

## Abstract

Here, we report a facile route for obtaining carbon spheres with fully tunable shell thickness. Using a hard template in chemical vapor deposition (CVD), hollow carbon spheres, solid carbon spheres, and intermediate structures can be obtained with optimized process time. The resulting carbon spheres with particle diameters of ~400 nm, as well as a controllable shell thickness from 0 to 70 nm, had high Brunauer–Emmett–Teller (BET) specific surface area (up to 344.8 m^2^·g^−1^) and pore volume (up to 0.248 cm^3^·g^−1^). The sphere formation mechanism is also proposed. This simple and reproducible technique can deliver carbon materials for various applications, e.g., energy storage and conversion, adsorption, catalytic, biomedical, and environmental applications.

## 1. Introduction

Carbon materials have been extremely popular for decades. Among them, carbon spheres attracted huge scientific attention [1]. Their history began in the 1990s. Firstly, solid and hollow carbon spheres were obtained from the thermal decomposition of methane [2,3,4] or camphor vapors [5] in the presence of a metal catalyst precursor. Currently, a number of techniques for preparing carbon spheres are applied, such as hydrothermal carbonization (HTC) [6], arc-discharge [7], and laser ablation [8]. However, the chemical vapor deposition (CVD) process [9] is the most common method for carbon sphere fabrication. Various precursors and templates were used to prepare carbon spheres using the CVD method. The templates were classified into hard and soft templates. Soft templates are precursors inducing the self-assembly of the final product, such as hexadecyltrimethylammonium bromide (CTAB). Hard templates are particles acting as solid cores with carbon replicas. Soft templates are much easier to be eliminated from the products. However, hard templates allow better control over the fabricated structures. Recently, aluminosilicate templates such as halloysite are becoming more and more popular. They can be used to obtain tubular structures [10]. However, to induce the formation of spherical carbon materials, silica spheres are the most commonly used templates. This is mainly due to a wide range of available diameters from 5 nm to several hundreds of nanometers, and the ease of the removal procedure from the final product [11,12,13]. Obtained hollow carbon spheres have various structural properties which result in high specific surface area, large pore volume, low density, thermal conductivity, and electrical conductivity, as well as good chemical and mechanical stability [14]. Additional unique properties of carbon spheres are related to low density, excellent reactivity, high compressive strength, thermal insulation, and large cavity space [15,16]. There are many reports describing the use of carbon spheres in energy storage and conversion, adsorption, catalytic, biomedical, and environmental applications. In the energy storage field, they are used as active materials of electrodes in supercapacitors [17,18,19] and lithium-ion batteries [20,21,22]. Furthermore, carbon spheres can act as catalysts in various reactions [23,24], and they are used for CO_2_ capture [25,26]. Moreover, they also serve well for biomedical applications [27,28,29], for example in drug delivery [27]. 

**Figure 1:**
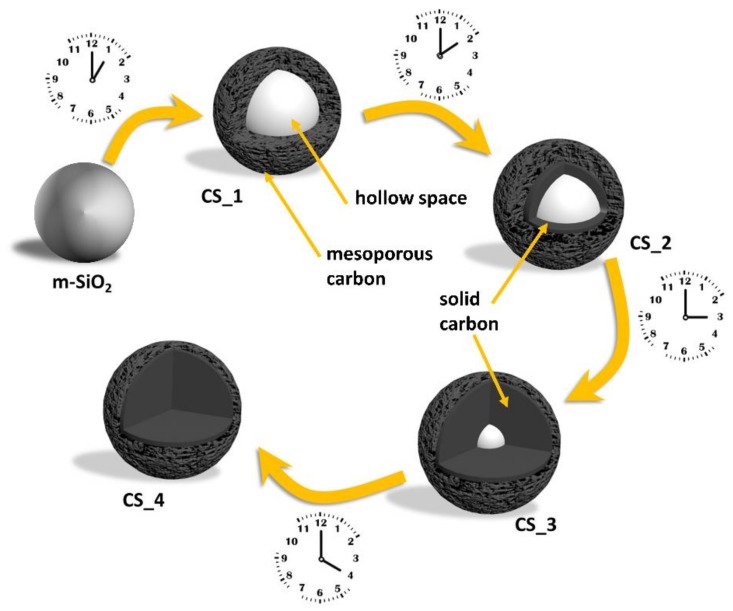
Scheme presenting the change in morphology of resulting carbon spheres after 1 h (CS_1), 2 h (CS_2), 3 h (CS_3) and 4 h (CS_4) of chemical vapor deposition (CVD).

So far, the control of the structure of carbon spheres is related to two main structural characteristics: (i) the shell thickness [30,31] and (ii) the order of the pores in the shell. The proposed carbon sphere can exhibit ordered [13,32] and disordered [33] pores. It is well known that the nanoparticle shape has a crucial role in its applications, such as drug delivery [34], filler for polymeric matrices [35,36], and deacidification [37,38]. In our contribution, solid silica nanoparticles serve as a hard template in CVD-grown carbon spheres. Furthermore, optimizing the process time of the procedure allowed the growth of carbon spheres with empty cores, solid carbon spheres, and intermediate carbon structures. We also propose a mechanism for sphere formation, taking into consideration the mesoporous silica particles (m-SiO_2_) being utilized as the template. Schematically, the change in morphology of the spheres prepared using CVD for different times, from hollow to solid carbon spheres, is shown in Figure 1.

## 2. Materials and Methods 

### 2.1. Synthesis of Mesoporous Silica Spheres

Mesoporous silica spheres were prepared as templates for further processing. Briefly, the surfactant, hexadecyltrimethylammonium bromide (CTAB, MERCK, Darmstadt, Germany; 900 mg), was added to a mixture of ethanol (EtOH, MERCK, Darmstadt, Germany; 180 mL), distilled water (240 mL), and ammonia (MERCK, Darmstadt, Germany; 25 wt.%, 3.3 mL), before being sonicated to obtain a homogeneous solution, and stirred vigorously for 30 min. Next, the silica precursor, tetraethyl orthosilicate (TEOS, MERCK, Darmstadt, Germany; 1.2 mL), was added to the reaction mixture and subsequently stirred at room temperature overnight. Finally, the product was centrifuged and dried [39].

### 2.2. Synthesis of Carbon Spheres with Different Shell Thickness (from Hollow to Solid Carbon Spheres)

The as-prepared m-SiO_2_ template was used to prepare the carbon spheres using the CVD method. The m-SiO_2_ template in an alumina boat was placed into a tube furnace in the presence of argon and ethylene at flow rates of 100 sccm and 30 sccm, respectively. The temperature was raised to 800 °C. Processes with different carbonization times (1, 2, 3, and 4 h) were performed. Afterward, the resulting spheres (m-SiO_2__CS) were washed with hydrofluoric acid to remove the silica and obtain the final product—carbon spheres with different morphology.

### 2.3. Characterization

The morphology of the samples was examined with a transmission electron microscope (TEM; Tecnai F30, Thermo Fisher Scientific, Waltham, MA, USA) and a scanning electron microscope (SEM; VEGA3 TESCAN, Brno, Czech Republic; high voltage (HV): 30 kV, working distance (WD): 5.25 mm). X-ray diffraction (XRD) patterns were carried out using an X’Pert Philips Diffractometer (X’Pert PRO Philips diffractometer, Co. Ka radiation, Almelo, Holland) with a Cu lamp (Kα1 = 1.54056 Å) to investigate the crystal composition of the samples. Thermogravimetric analysis (TGA) was carried out on 10-mg samples using a DTA-Q600 SDT TA Instrument (TA Instrument, New Castle, DE, USA) at a heating rate of 5 °C/min from room temperature to 900 °C in air. Raman spectra were determined using an inVia Raman Microscope (Renishaw, New Mills Wotton-under-Edge, UK) with an excitation wavelength of 785 nm. N_2_ adsorption/desorption isotherms were obtained using a Quadrosorb SI (Quantachrome Instruments, Boynton Beach, FL, USA). Specific surface area was calculated according to the Brunauer–Emmett–Teller (BET) method, and pore size distribution was determined using the density functional theory (DFT) method.

## 3. Results and Discussion

**Figure 2:**
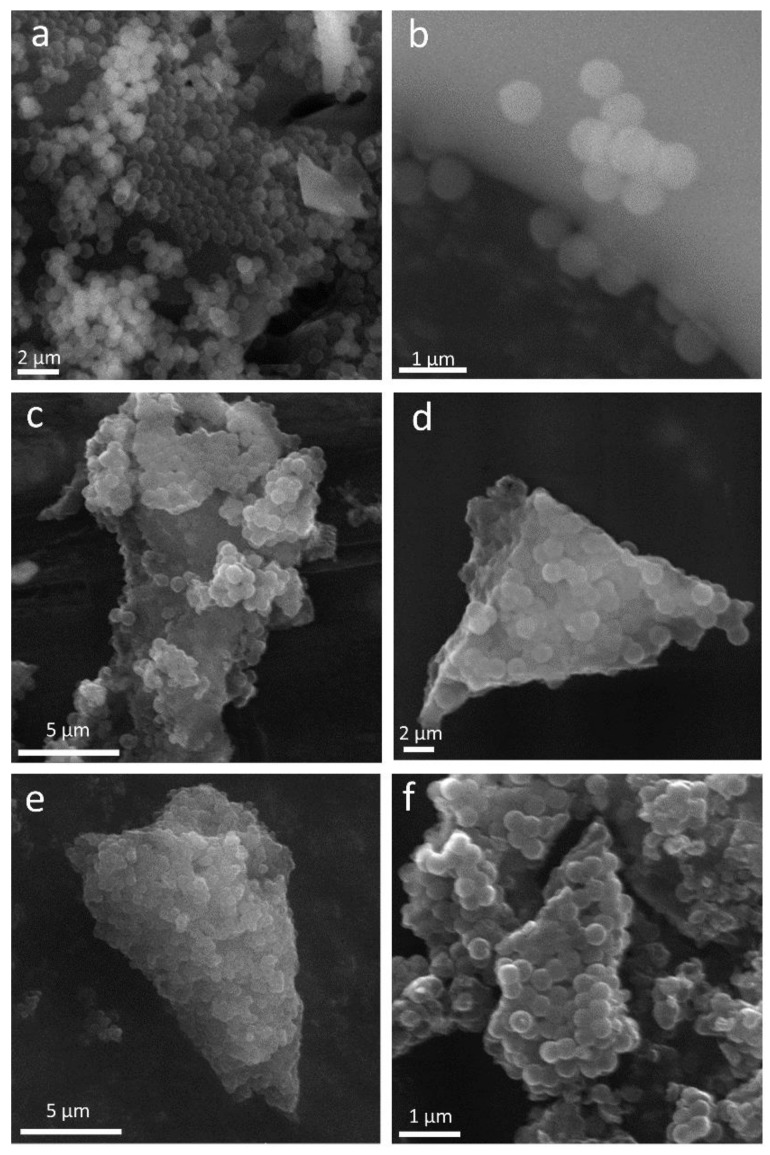
SEM images of silica template (**a**,**b**) and carbon spheres after 1 h (**c**), 2 h (**d**), 3 h (**e**), and 4 h (**f**) of synthesis.

Scanning electron microscopy was used to reveal the evolution of morphology and structure of the obtained materials, i.e., the silica template and all carbon spheres. As illustrated in Figure 2a,b, a uniform and spherical shape with a diameter of ~400 nm, as well as a smooth surface, can clearly be observed in the silica template sample. After the CVD process, carbon materials kept the initial spherical shape of the silica template and exhibited a similar diameter of ~400 nm. It can clearly be observed that, with an increase in synthesis time, the morphology of the carbon spheres did not change significantly (Figure 2c–f).

**Figure 3:**
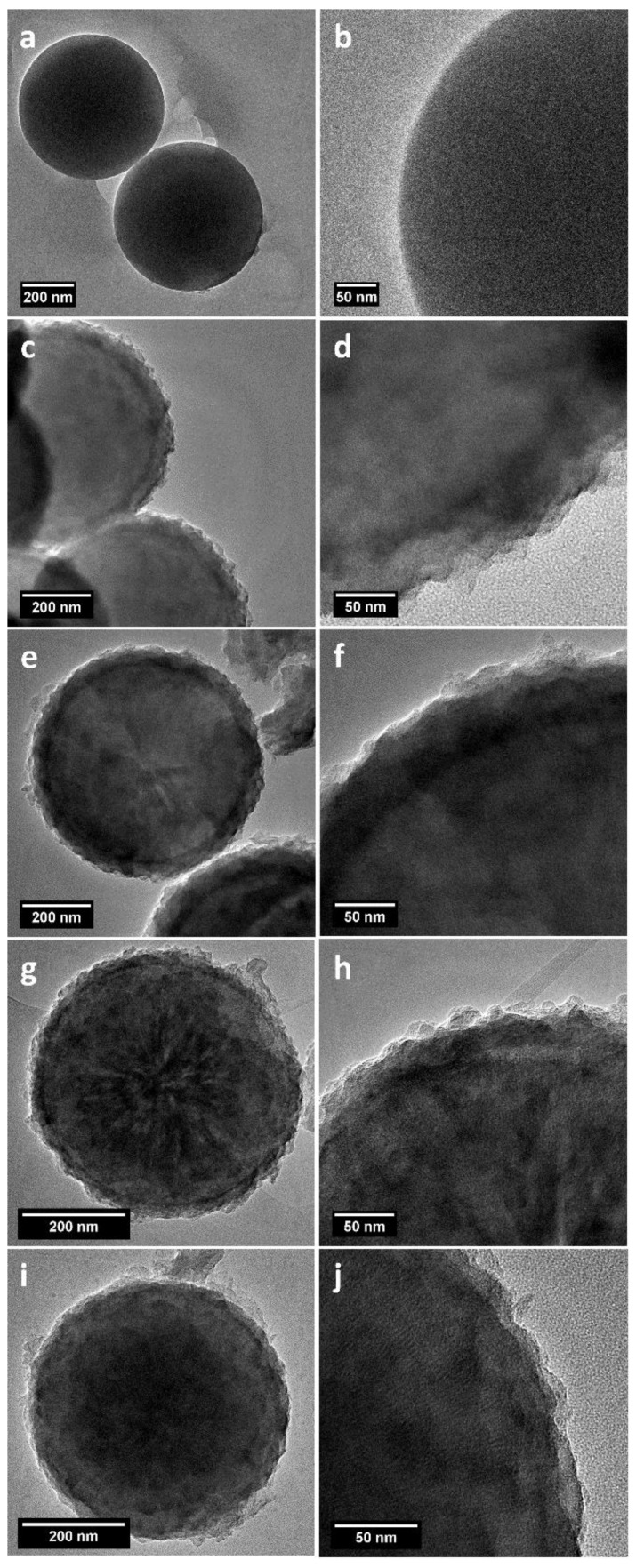
TEM images of silica template (**a**,**b**) and carbon spheres after 1 h (**c**,**d**), 2h (**e**,**f**), 3 h (**g**,**h**), and 4 h (**i**,**j**) of synthesis.

Representative TEM images of silica spheres and carbon spheres are presented in Figure 3. Silica spheres used as a template for the carbon spheres had diameter of ~400 nm (Figure 3a,b), which was confirmed by SEM analysis [34]. The carbon spheres after removal of the silica template had porous shells with thicknesses changing with time. After 1 h (CS_1) of CVD, the shell thickness was ~70 nm (Figure 3c,d). After 2 h (CS_2), the shell diameter decreased to ~30 nm (Figure 3g,h). The spheres produced after 3 h of CVD (CS_3) had a shell thickness ~20 nm (Figure 3i,j). When the process took 4 h, the sample did not exhibit the presence of a shell. Its diameter was the same as the silica template. Moreover, the core of the sphere was no longer hollow. Solid carbon spheres were produced under these experimental conditions. Interestingly, with the increase in process time, the carbon atoms increasingly diffused from the external shell into the spheres.

**Figure 4:**
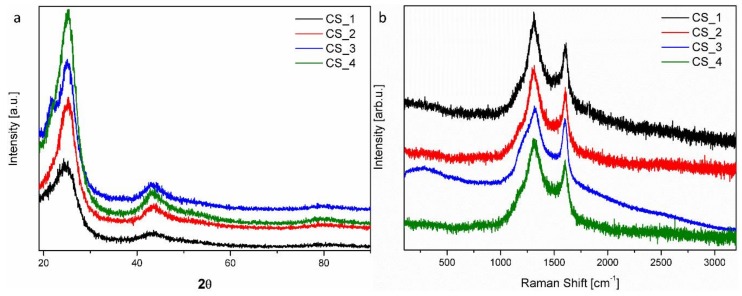
X-ray diffraction (XRD) patterns (**a**) and Raman spectra (**b**) of carbon spheres with different times of synthesis.

The graphitization degree of the carbon spheres was determined using XRD. Figure 4a displays the XRD patterns, in which the two peaks at ~25° and ~43° can be assigned to typical graphitic (002) and (100) planes, respectively. The broadening of the two peaks suggests a low graphitization degree and the possible presence of amorphous carbon. However, upon extending the CVD process time, sharper peaks with greater intensity were detected. This means that the degree of graphitization of the final material increased.

The bonding, order, and crystallinity of the materials were studied using Raman spectroscopy (Figure 4b). The presence of disordered graphitic materials was suggested by the two Raman modes. The peak at 1604 cm^−1^ (G band) corresponds to the E_2_g mode of hexagonal graphite, and it is related to the vibration of the sp_2_-hybridized carbon atoms in the graphite layer. This implies that the carbon spheres were composed of graphitic carbon, which is consistent with the TEM and XRD results. The D band at approximately 1312 cm^−1^ is associated with the vibration of carbon atoms with dangling bonds in the plane with termination by disordered graphite. The D band had higher intensity than the G band, which suggests that the obtained carbon spheres had several defects, and they consisted mostly of amorphous carbon [40].

**Figure 5:**
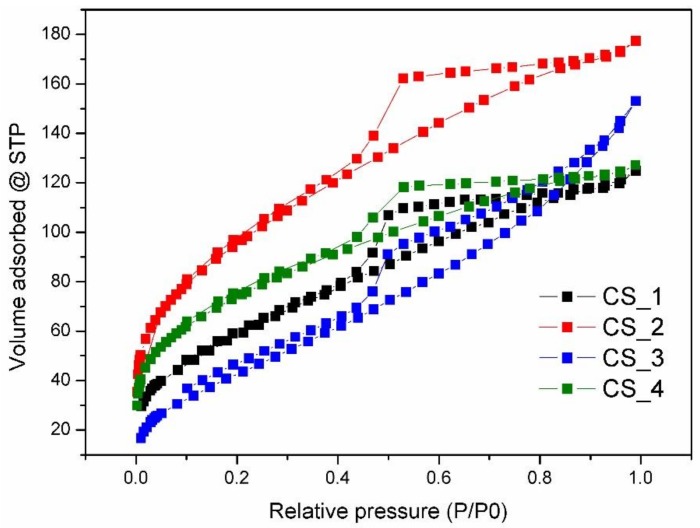
N_2_ sorption isotherms of carbon spheres with different times of synthesis.

The porosity of the synthesized samples was tested using N_2_ adsorption/desorption experiments. The textural properties are listed in Table 1. Type IV isotherms with H4 hysteresis loops were observed in all samples, which are typical of mesoporous materials (Figure 5). The position of the P/P_0_ inflection points is associated with the range of mesopore size, and the slope degree of the steps indicate the uniformity of mesopore size. There were capillary condensation steps at P/P_0_ of 0.4–1.0, ascribed to mesopores in the samples [41]. The pore size distribution curves show the existence of uniform mesopores below 4 nm. These mesopores should be located in the shell of carbon spheres. Upon increasing the time of synthesis, the BET specific surface area of carbon spheres increased gradually to 344.8 m^2^·g^−1^, as well as the total pore volume to 0.248 cm^3^·g^−1^, in CS_2. A further increase in the process time caused a decrease in BET specific surface area and total pore volume, which may be related to the blocking of mesoporous channels by the diffused carbon. When the carbon spheres were filled with carbon, the diffusion ceased and the shell disappeared; the BET specific surface area and total pore size distribution were again enhanced. The control of the texture parameters of carbon spheres, such as specific surface area, total pore volume, and pore size, is important in adsorption and catalytic applications.

**Figure 6:**
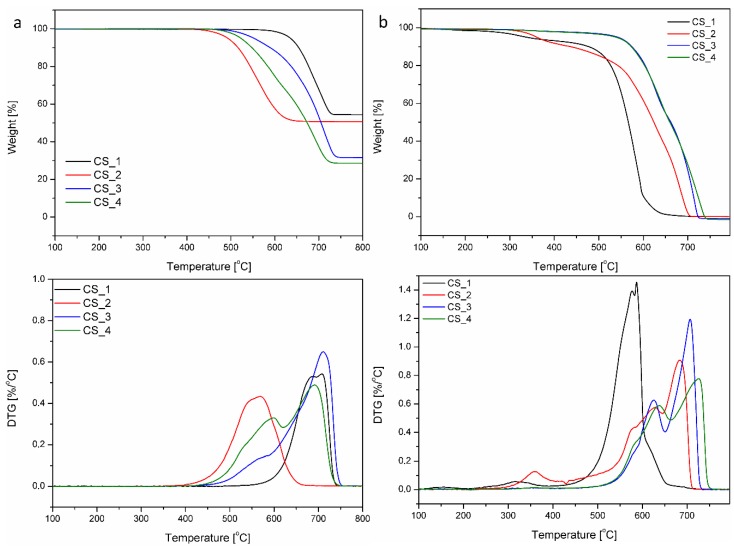
Thermogravimetry analysis (TGA) and differential thermogravimetry (DTG) curves of carbon spheres with different times of synthesis. Before (**a**) and after (**b**) removing silica template.

The TGA measurements in Figure 6 provide information on the carbon content and quality of the structure in the carbon spheres. It is known that carbon with a better crystalline structure decomposes at higher temperature. For example, carbon nanomaterial with well-ordered sp_2_ hybridization starts decomposing above 600 °C [42,43,44], while amorphous carbon initiates its decomposition at a lower temperature, ca. 500 °C and below [45,46]. Figure 6a shows that our carbon spheres were oxidized above 415 °C, which means that our carbon spheres consisted mostly of amorphous carbon, which is in full agreement with the Raman and XRD spectra. As the temperature increased further, weight decreased rapidly until all of carbon spheres were exhausted at approximately 730 °C. The ash contents of the samples after combustion were 54.3% (*w*/*w*) for m-SiO_2__CS_1, 50.6% for m-SiO_2__CS_2, 31.5% for m-SiO_2__CS_3, and 28.5% for m-SiO_2__CS_4, which indicates that the contribution of silica decreased. A simultaneous increase in synthesis time and the amount of carbon in the samples was observed. After removing the silica core, the TGA curves changed slightly (Figure 6b). All samples consisted only of carbon; thus, they were completely burnt at high temperatures of up to 800 °C, proving the high purity of the final samples. However, the temperature of total combustion was different, and it shifted with the time of the CVD process. CS_1 burned completely at about 650 °C, while CS_4 burned completely at about 755 °C. Full thermal decomposition parameters calculated from the differential thermogravimetry (DTG) curves are listed in Table 2. The start (T_start_) and end (T_end_) temperatures of the peak, as well as the temperature at which the peak had its maximum (T_max_), are specified. Both T_max_ and T_end_ shifted to higher temperature values with an increase in CVD process time. This indicates that the carbon crystallinity was enhanced when the CVD process was longer. This is in good agreement with the increasing intensity of the peak at 25° in the XRD measurements shown earlier.

Detailed microscopic analysis allowed a proposal of the growth mechanism. In the initial phase of synthesis, carbon atoms adsorbed onto the surface of SiO_2_, forming a porous shell. Therefore, the diameter of the spheres after 1 h of CVD process was larger than that of the pristine template. With an increase in CVD time, carbon atoms started diffusing from the external part of the shell to the core, and the shell thickness decreased. However, more carbon atoms were deposited onto the interior of the template. Gradually, the shell disappeared and, at some point, the mesoporous channels of silica were filled with diffused carbon. When the carbon spheres were filled with carbon, the diffusion slowed down and the shell disappeared, resulting in the reduction in BET specific surface area and pore size distribution of the carbon spheres. This effect was clearly observed in the case of CS_4.

## 4. Conclusions

In this work, we successfully fabricated solid and hollow mesoporous carbon spheres with controllable shell thickness, as well as high specific surface area and pore volume, using the CVD method. Specific surface areas and pore volume distributions of the carbon spheres could be tuned with the CVD process time. Optimizing the process time of the procedure allowed the growth of carbon spheres with empty cores (1 h CVD), solid carbon spheres (4 h CVD), and intermediate carbon structures (2 or 3 h CVD). The mechanism of sphere formation was also proposed. It is believed that this facile synthesis route can be a way of preparing carbon spheres with different morphology (from hollow to solid), which can be tested for various applications, such as energy storage and conversion, adsorption, catalytic, biomedical, and environmental applications.

## Figures and Tables

**Table 1:** Texture parameters of carbon spheres with different times of synthesis. SBET—Brunauer–Emmett–Teller surface area; Vtotal—total pore volume; m-SiO_2_—mesoporous silica; CS_1–4—carbon spheres with 1–4 h of chemical vapor deposition.

Sample	SBET ^a^ (m^2^·g^−1^)	Vtotal ^b^ (cm^3^·g^−1^)	Pore Size ^c^ (nm)	Error ^d^
m-SiO_2_	224.2	0.210	2.313	0.391%
CS_1	218.2	0.174	3.969	0.609%
CS_2	344.8	0.248	3.167	0.970%
CS_3	169.8	0.212	2.245	0.544%
CS_4	263.5	0.177	1.178	0.621%

^a^ Determined using the multipoint BET method. ^b^ Calculated from density functional theory (DFT) method for cumulative pore volume. ^c^ Determined by the DFT pore diameter mode. ^d^ Fitting error from the DFT method summary.

**Table 2:** Thermal decomposition parameters of carbon spheres with different times of synthesis from differential thermogravimetry (DTG) curves in Figure 6b.

Sample	T_start_ (°C)	T_max_ (°C)	T_end_ (°C)
CS_1	110	580	650
CS_2	220	680	715
CS_3	450	705	735
CS_4	450	725	755
